# Comparative Two-dimensional Gel Electrophoresis Maps for Amastigote-like Proteomes of Iranian *Leishmania Tropica* and *Leishmania Major* Isolates

**DOI:** 10.31661/gmj.v8i0.1520

**Published:** 2019-08-15

**Authors:** Marzieh Ashrafmansouri, Fatemeh Sadat Sadjjadi, Seyyedjavad Seyyedtabaei, Ali Haghighi, Mostafa Rezaei-Tavirani, Nayebali Ahmadi

**Affiliations:** ^1^Student Research Committee, Department of Medical Parasitology and Mycology, School of Medicine, Shahid Beheshti University of Medical Sciences, Tehran, Iran; ^2^Proteomics Research Center, Faculty of Paramedical Sciences, Shahid Beheshti University of Medical Sciences, Tehran, Iran; ^3^Department of Medical Parasitology and Mycology, School of Medicine, Shahid Beheshti University of Medical Sciences, Tehran, Iran

**Keywords:** Leishmaniasis, Proteomics, Leishmania Major, Leishmania Tropica, Two-Dimensional Gel Electrophoresis

## Abstract

**Background:**

*Leishmania major* and *Leishmania tropica* are the main causative agents of cutaneous leishmaniasis. Proteomics as a novel approaches could be used to evaluate protein expression levels in different stages of *Leishmania* species. We compare the protein contents of amastigote-like forms in *L. tropica* and *L. major* using two-dimensional gel electrophoresis (2-DE) and bioinformatics methods.

**Materials and Methods:**

*Leishmania* parasites were isolated from the lesions of Iranian patients and identified using restriction fragment length polymorphism-polymerase chain reaction (RFLP-PCR). Five isolates of each two species were cultured in specific media to obtain amastigote-like forms to be prepared for proteomics study. Total protein contents were separated using 2-DE. The gels were stained by silver nitrate and scan was imaged. The protein spots with different expression changes in each gel were analyzed using Progenesis SameSpots software.

**Results:**

A total of 354 protein spots were detected in both amastigote-like forms. Comparative analysis of protein spots with different expressions in the two amastigote-like form species showed 173 highly expressed spots of which 74 *L. tropica* and 99 *L. major* proteins were spotted with fold≥2. Also, 16 and 20 new protein spots were uniquely found in *L. tropica* and *L. major*, respectively. Clustering of different detected proteins using correlation analysis divided the proteins into two clusters based on their expression level. Furthermore, clustering results were confirmed by principal component analysis.

**Conclusion:**

Using proteomics methods specially 2-DE and statistical analysis demonstrated significant changes in protein expression levels in amastigote-like forms of *L. tropica* and *L. major* isolates.

## Introduction

*Leishmania* as the protozoan intracellular parasites of the Tripanosomatidae family that causes a group of diseases named leishmaniasis with different clinical forms, from simple self-limiting cutaneous scars to severe visceral leishmaniasis [1, 2]. Cutaneous leishmaniasis (CL) is endemic in many areas of the world including Middle-East. In the region, *L. major* and *L. tropica* are the main causative agents of zoonotic and anthroponotic CL, respectively[3, 4]. *Leishmania* parasites have two recognizable stages in their life cycle, promastigote and amastigote [5, 6]. In the promastigote stage, the parasite grows in sandfly vector while they are flagellated and spindle-shaped. When the parasite is deployed inside the parasitophorous vacuole of mammalian macrophages differentiated to the amastigote stage, the parasite is non-motile and flagel free. Amastigotes change their protein/gene expression levels to adapt themselves with the environment inside the macrophages, and starts causing the disease [6, 7]. Therein the submembrane microtubule can find a new arrangement, the consumption of oxygen is minimized, and the metabolic pathways related to the consumption of host nucleic acid precursors are activated. Amastigotes are resistant to digestion by lysosomal enzymes, while promastigotes are susceptible to these enzymes and some of them disappear within the macrophage before they transform to the amastigote [8, 9]. Since the culture of promastigotes at in vitro is easy to accomplish, in recent years, extensive researches on *Leishmania* parasite have been done using in vitro culture promastigotes [10], while the amastigote stage is the cause of disease in humans. Addressing this problem, the amastigote-like of *L. tropica* and *L. major* are investigated in this study to find the similarities and differences of these two types of *Leishmania* in terms of characteristics such as surface proteins. It can be effective in concluding in vitro tests and the design of new therapies [11, 12]. Knowing the differences and antigenic similarities between amastigote-like of *L. tropica* and *L. major* is specifically effective in the development of vaccines that are based on antigenic components. Identifying the protein content of various forms of the parasite can help to understand the differences in enzyme pathways, metabolic processes, protein function, protein level, and its modification, which facilitate evaluating biological or biochemical roles of proteins and improving the drug design [11, 13, 14].Two-dimensional gel electrophoresis (2-DE) technique is one of the common proteomics methods which can separate proteins in first and second dimensions according to their isoelectric points (PI) and molecular weight. Previous proteomic analysis has been focused mainly on promastigote stages from among the different stages present in the *Leishmania* lifecycle [6, 15-17]. Since *L. tropica* and *L. major* are endemic in Iran, and there is no data on the proteome assessment of amastigotes of Iranian *Leishmania* isolates, the present study for the first time was conducted to compare the protein spot contents of amastigote-like forms in these two species using 2-DE and bioinformatics methods.

## Materials and Methods

### Sampling, Culture, and Identification

This study was carried out on clinically suspected cases of CL referred to the health centers of Mashhad, Gonbad, Kerman, Shiraz, and Tehran cities during 2016-2017. This study was approved by the Ethics Committee of Shahid Beheshti University of Medical Sciences (approval code: IR.SBMU.MSP.REC.1395.351), official informed consent was taken from all of the participants. For parasitological diagnosis, smears were prepared through sampling the border of skin lesions of each suspected case, and then microscopically checked for amastigote stage. Positive skin scrapings were cultured in Novy-MacNeal-Nicolle medium at 24°c for ten days (range 1-2 weeks). Extracted and purified DNA was used to perform polymerase chain reaction (PCR) for identifying the internal transcribed spacer 1 (ITS1) gene of the *Leishmania* Spp. Restriction fragment length polymorphism (RFLP) method using *Hae* III (Fermentas, Leon-Rot, Germany) enzyme was performed to differentiate* L. major* from *L. tropica* according to banding patterns of standard *Leishmania* species[18]. A total of ten PCR-RFLP confirmed positive samples (five *L. major* and five *L. tropica*) were used for later stages of research. The promastigotes were transferred into RPMI-1640 medium (Gibco, Germany), (PH=7.1) containing 10% heat-inactivated fetal calf serum (Gibco, Germany), 100ug/ml streptomycin (Gibco, Germany), 100U/ml penicillin (Gibco, Germany), incubated at 25°C with adding the fresh media to the grown cultures twice a week. The mass cultures examined by invert microscope and grown parasites were checked by light microscope.

### Preparation of Amastigote-Like Forms

In order to convert promastigote form to amastigote-like form, the environmental condition was changed. Promastigotes collected in the stationary phase by centrifugation at 3000rpm, 4°C and 20 minutes, after that they were washed in sterile phosphate buffered saline (PBS), PH=7.2-7.4. Then followed by adding RPMI1640 medium, which was previously adjusted the PH to 3.5-4 by adding sterile 10 mM succinic acid (Merck, Germany), supplemented with 20-25% fetal calf serum, 200U/ml penicillin and 200ug/ml streptomycin (Gibco, Germany). Furthermore, we incubated the cultured media at 35 °C with 5% CO2 for 96–120 hours. Afterthat the media were checked every day for any changes in cultivated parasites through Giemsa (Merck, Germany) staining and light microscopic observation. The amastigote-like formation was confirmed by observing flagellum and cell roundness and then subsequently harvested by centrifugation at 2500rpm, 10 minutes and washed three times in sterile phosphate buffered saline (PBS, PH=4.5). A cell suspension containing 10^7^ parasites/ml was collected and then stored at -70°C until further use.

### Protein Extraction

The amastigote-like of parasites were washed in sterile PBS (PH=7.2-7.4). Then it is resuspended in Tris- HCl (Roche, Germany) with PH=7.8 and 5 mM concentration. Tris- HCl contains a protease inhibitor (Roche, Germany), 1mM phenyl methyl sulfonyl fluoride. Afterward, the samples were sonicated 3 times at 40 Hz for 10 seconds with 50 seconds intervals. The samples are kept for 4 hours at 4°C. In order to have precipitated proteins, 20% trichloroacetic acid with 20 mM dithiothreitol (Merck, Germany) were used at -20°C for 1h. Then samples were centrifuged with the angular speed of 13000 rpm for 15 minutes at 4°C. It washed with the cold acetone solution of 20 mM dithiothreitol; the samples were air-dried overnight and dissolved in 100 μl lysis buffer. Using the Bradford method, Protein concentration was determined with bovine serum albumin standard.

### The 2-DE Assay and Staining

Firstly, electrophoresis was performed on the 7 cm strips of immobilized PH gradient (IPG), pH=4-7 (Bio-Rad, Hercules, CA), which were rehydrated by loading the samples with rehydration buffer over-night. After the gel rehydration, isoelectric focusing was performed at several voltages and times, and then IPG strips were incubated in two equilibration solution with DTT and iodoacetamide, respectively. The strips that were placed on top of 12% gradient SDS-PAGE were sealed with agarose solution (Bio-Rad, USA). The 2-DE method was performed on 16 mA/gel and 24 mA/gel at 20 °C for 30 minutes until the dye front reached the lowest part of the gel. Finally, gels were stained by fresh silver- nitrate [19].

### Statistical Analysis

The 2-DE gels were scanned by densitometer GS-800 (BioRad, USA) and prepared for analyzing of the gel images using Progenesis SameSpots software, version 4.0 (nonlinear dynamics: A water company). The Progenesis SameSpots software controls the quality of gels images, compares gels and protein spots detection, color intensity assessment of spots, spots alignment and carries out the statistical analysis. For determining significant statistical changes in proteins expression, analysis of variance was performed, and fold changes were calculated. Finally, protein spots with fold≥2 and P-value<0.05 selected as significant spots. Hierarchical clustering was used for comparing proteins between *L. major* and *L. tropica* and principal component analysis (PCA) was carried out for clustering confirmation.

## Results

**Figure 1:**
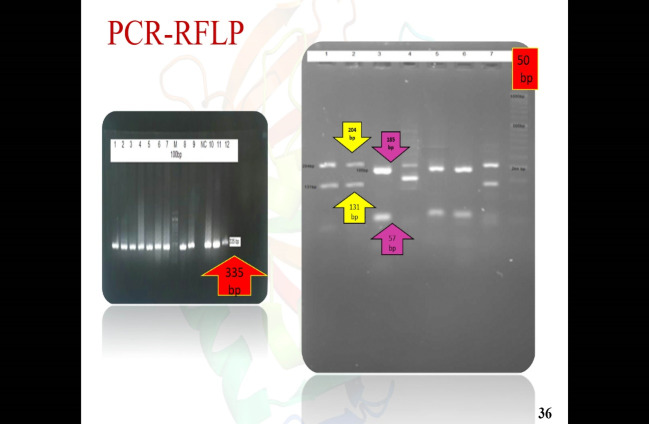
Restriction enzyme digestion profile of amplified ITS1region form *Leishmania* cultures with the enzyme *Hae* III. Molecular marker (50 bp);* L. tropica* (fragments 57 bp, 55 bp, 20 bp and 185 bp) that two bands (55bp, 20bp) are visible under the influence of UV rays;* L. major (* fragments 131 bp and 204 bp); **Lane1:** Standard *L. major*; **Lane 3:** Standard *Leishmaniatropica*; **Lane 2, 4, 7:** *L. major* from the samples of patients; **Lane 5, 6:** *L. tropica* from the samples of patients.

**Figure 2:**
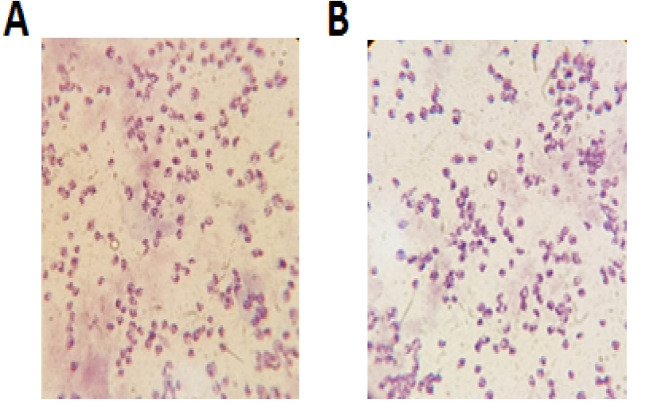
Optical microscopy images of amastigote-like forms of *Leishmaniatropica* (**A**) and *L. major*(**B**).The cells were stained with Giemsa and studied by light microscopy, using1000× magnification

**Figure 3:**
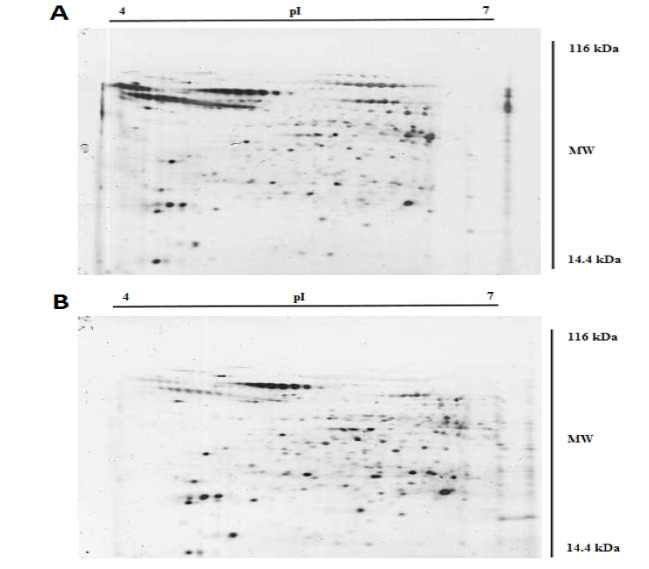
The 2-DE gel images of amastigote-like forms proteins of *Leishmaniatropica* (**A**) and *L. major*(**B**). Proteins were separated on 7 cm IPG strips (PH=4-7) in the first dimension. The second dimension of 2-DE was performed using SDS-PAGE 12%. The gels were stained by silver nitrate followed by scan imaging of the gels.

**Figure 4:**
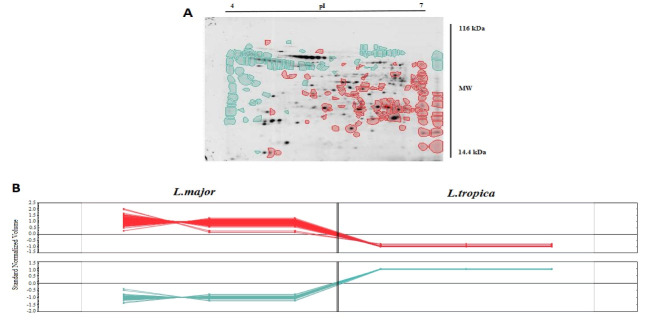
**A:** Representation of protein spots with a significant difference in their expression in amastigote-like forms of *Leishmania tropica* (74 spots in blue color) and *L. major* (99 spots in red color). Blue spots are highly expressed proteins in *L. tropica,* and red spots showed high expressed proteins in *L. major*. **B:** The diagram showed protein expression trends in two groups. Each line is representative of each protein spot.

**Figure 5:**
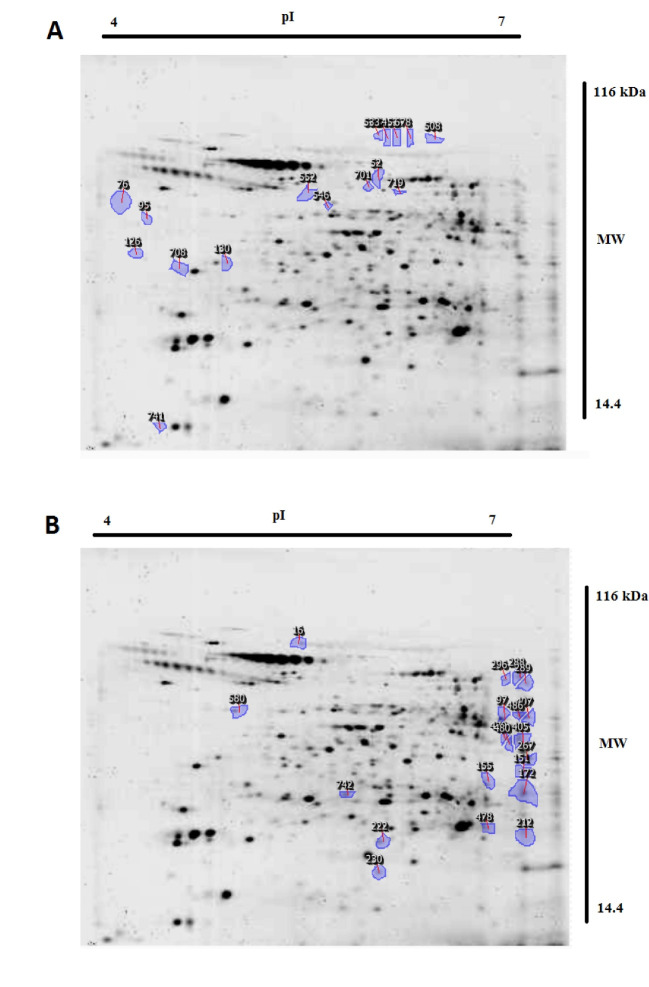
Representative of 16 unique spots in *Leishmaniatropica* (**A**) and 20 spots in *L. major*(**B**). Images showed that specific proteins in* L. tropica* are in all region of gel, but in* L. major* the most specific proteins are present in the near neutral area.

**Figure 6:**
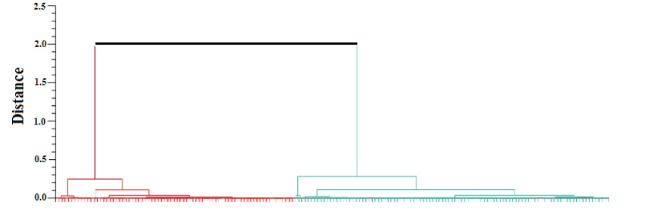
Hierarchical clustering of protein spots. The diagram indicates the cluster of 99 protein spots which found with high expression in*Leishmania major* (red) and 74 protein spots found with high expression in* L. tropica* (blue). All of the proteins with different expression have been classified into two groups: either high expression and low expression. It should be noted that highly expressed proteins in *L. major* isolate are the same as low expressed proteins in* L. tropica* isolates and vice versa.

**Figure 7:**
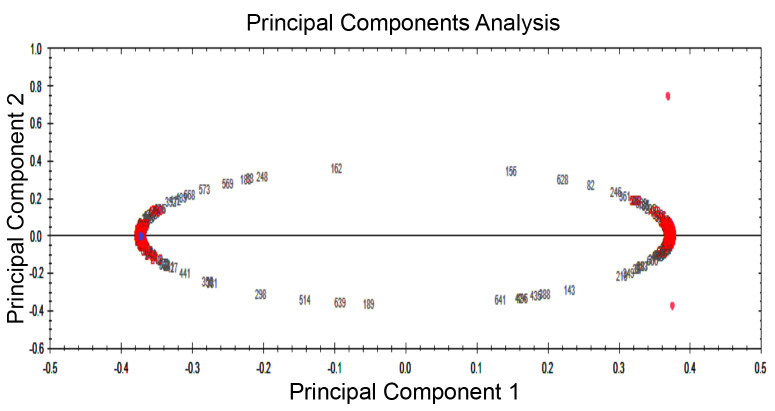
Principal component analysis (PCA) was performed. The PCA of 74 protein spots were highly expressed in*Leishmania tropica* (left) and 99 protein spots were highly expressed in* L. major* (right). It should be noted that highly expressed proteins in* L. major* isolate are the same as low expressed proteins in *L. tropica* isolates and vice versa.

PCR resulted in the amplification of the *Leishmania* strains, samples giving 340 bp amplification bands. Restriction of the ITS1 gene amplicons of *L. major* with the endonuclease *Hae* III generated patterns with two bands of 204 and 131 bp; in *L. tropica* 185 and 57 bp and two other bands (55bp, 20bp) are visible under the influence of ultraviolet rays (Figure-1).Amastigote-like forms of *L. major* and *L. tropica* were produced from in vitro transformation of promastigote forms of *L. major* and *L. tropica*. The optical microscopy images of amastigote-like forms of *L. major* and *L. tropica* indicated in Figure-2. The amastigote-like formation is confirmed by observing cell roundness, a flagellated and immobile forms. The 2-DE technique was used for separating the whole proteome in amastigote-like form of Iranian *L. tropica* and *L. major* isolates. The gels were stained with silver nitrate followed scanned by a densitometer and prepared for analyzing. A total of 354 protein spots have been detected in (Figure-3). Among these spots,173 protein spots with different expression were detected in the amastigote-like proteome of *L. tropica* and *L. major* by analyzing gels using Progenesis SameSpots software. The Comparison of protein spots between amastigote-like forms of* L. tropica* and *L. major* isolates showed that 74 spots had high expression in amastigote-like form of *L. tropica* relative to amastigote-like forms of *L. major*. On the other hand, 99 proteins spots had high expression in amastigote-like forms of *L. major* relative to amastigote-like forms of *L. tropica* (Figure-4). Among these spots, it observed that 16 specific proteins are present in amastigote-like forms of *L. tropica* while they were not seen in amastigote-like forms of *L. major*. On the other hand, gels analysis showed that 20 specific proteins are present only in the amastigote-like form of *L. major* isolate (Figure-5). Characteristics of unique protein spots in each isolate represented in Table-1. Hierarchical clustering of protein spots with different expression of amastigote-like forms of *L. tropica* and *L. major* isolates performed using correlation analysis. Cluster analysis divided proteins into two subgroups based on their expression status which showed in Figure-6. Proteins that are correlated in each cluster suggest that these proteins may have similar function or involved in common biological pathways. PCA was performed to confirm the clustering of expressed proteins of amastigote-like forms of *L. tropica* and *L. major* isolates (Figure-7).

## Discussion

A proteomics approach for investigation on many aspects of leishmaniasis is a new tool, which could be provided a wide range of data set. Proteomics studies of *Leishmania* parasites could lead to the identification of specific protein markers of each strain that are responsible for the pathogenesis of the parasite [20]. In vitro culture of promastigote forms of *Leishmania* species is easy. Therefore, in recent years, a lot of research on *Leishmania* has been carried out using *in vitro* culture of promastigotes. But promastigote form is a form of parasite which grows and proliferates in the body of sand flies while amastigote form is a form of parasite that is located on the scar on the vertebrate host and it causes the disease. So the results of research on promastigote forms can’t be generalized to amastigote forms [12]. The present study attempted to differentiated promastigote forms of Iranian *L. tropica* and *L. major* isolates to amastigote-like forms in culture media and evaluated the protein expression levels in each isolate. In this study, amastigote-like forms generated successfully that similar to study performed by Habibi and her colleagues [5]. Proteomics tools and statistical analysis on *Leishmania’s* proteome of Iranian isolates and comparing their protein expression levels could help the investigators for more reliable diagnosis and treatment of the disease [19, 21]. Some studies have been conducted on proteomic profiling for virulence factors in humans and bacteria, but there are only a few reports for profiling parasite proteins [22], so the proteomes of many parasites are still unknown. In this regards, we performed a proteomics study on amastigote-like forms of Iranian *L. tropica* and *L. major* isolates using 2-DE. The protein maps of two isolates were compared which showed 173 protein spots with a different expression that 74 and 99 protein spots had significant high expression in amastigote-like forms of *L. tropica* and *L. major* respectively. Among these different proteins, 16 and 20 new protein spots are unique in *L. tropica* and *L. major* respectively. These specific proteins in *L. tropica* are in all region of gel, but in *L. major* the most specific proteins are present near the neutral area of gel (Figure-5). This shows that different proteins especially unique proteins of *L. tropica* and *L. major* are responsible for creating a specific clinical phenomenon. A similar finding has been reported for comparing proteome of promastigotes of *L. amazonensis* and *L. major* using 2-DE [16]. Brobey *et al*. using by 2-DE pattern detected that 120 proteins of promastigote form in the *L. amazonnesis* were upregulated compared to *L. major* [16]. Application of the 2-DE method for protein separation of amastigote-like forms of Iranian *L. tropica* and *L. major* isolates has also been used for another leishmaniasis isolates in promastigote stage including *L. amazonensis, L. braziliensis* and *L. major* [16, 17]. On the other hand, the 2-DE method was applied for the detection of proteins that respond to drug resistance during leishmaniasis treatment [21]. The molecular weights of all detected proteins were 14.4 to 116 kDa which is close to Zarean and his colleagues study that they reported most of these proteins to have significant roles in transferring other proteins, heat shock proteins, skeletal proteins, and hypothetical proteins, specifically they have protective roles for DNA structure and normal metabolism in the cells [21, 23, 24]. Among the expressed proteins, specific proteins of each isolate may be secreted to patients’ skin, which can be introduced as responsible protein/s for the clinical phenomenon of disease. To determine the pathogenesis of the disease, protein identification with mass spectrometry or shut-gun proteomics is necessary [25]. Separated proteins in the amastigote-like forms of *L. tropica* and *L. major* have been analyzed using Progenesis SameSpots software. The software performs clustering and correlation analysis in many groups. Cluster analysis is a method to discover the biomarkers, which are a heterogeneously populated divide within some homogeneous subsets that is called a cluster. Also, it is used to find groups that are very different from each other, but their members are very similar [26, 27]. This part is similar to the works on hydatidosis patients’ sera in human and Alzheimer disease in rat hippocampus proteome, which the authors reported that each cluster may have similar function or common signaling pathways [13, 28]. In order to analyze the data statistically, the PCA technique carried out to detect the variation of the data and help to visualize data easily. PCA is usually used for outlier data to confirm data clustering. PCA was used to assess the reliability of the classification of samples to different groups. In this study, PCA was applied to data analysis, and it showed no outlier in protein data set of both groups. PCA has been already applied to classify the metabolomics and proteomics data set of *Leishmania* to classify its proteins and metabolites [6, 11]. We observed several technical limitations, especially the inefficiency in identifying membrane proteins, which are major targets for therapeutic compounds [16]. Other technologies are necessary for a complete representative proteome such as mass spectrometry approach and Western blot detection system.

## Conclusion

To our best of knowledge, this is the first study on *L. tropica* amastigote-like from Iranian patient samples. We show the amastigote-like forms of *Leishmania* can be used instead of amastigote itself. Our findings showed significant differences in the protein content of amastigote-like forms between* L. tropica* and *L. major* isolates using the 2-DE technique. Application of proteomics methods could help to design new drug targets or new vaccination targets. To determine the molecular mechanisms of specific proteins in amastigote-like forms of *L. tropica* and *L. major* isolates it is needed to clarify the types of proteins by more researches.

## Acknowledgment

The authors gratefully acknowledge the financial support from the Proteomics Research Center, and Vice-Chancellor for Research at Shahid Beheshti University of Medical Sciences (grant number: 14519). This work is a part of the Ph.D. thesis of Marzieh Ashrafmansourifrom Parasitology and MycologyDepartment of Shahid Beheshti University of Medical Sciences.

## Conflict of Interest

The authors declared no conflict of interest.

**Table 1:** Characteristics of Unique Protein Spots That Detected In Leishmania major and L. tropica

**Spot No.**	**Species**	**ANOVA (p)**	**Fold**	**PI**	**MW**
296	*L. major*	1.52E-05	3	6.72	99
405	*L. major*	1.95E-06	3.7	6.82	80
407	*L. major*	3.21E-06	10.3	6.86	87
267	*L. major*	6.93E-07	5.9	6.87	73
288	*L. major*	1.68E-05	4	6.82	100
289	*L. major*	5.10E-06	5.9	6.87	98
486	*L. major*	5.92E-07	5.2	6.82	88
580	*L. major*	2.48E-09	2.9	4.97	89
742	*L. major*	4.21E-08	2.7	5.69	61
478	*L. major*	1.49E-06	2.9	6.58	50
479	*L. major*	0.005	4.3	6.72	80
480	*L. major*	1.36E-06	8.8	6.76	79
230	*L. major*	5.71E-07	2.2	5.89	36
151	*L. major*	6.28E-05	3.5	6.82	69
97	*L. major*	7.72E-13	4.5	6.69	87
16	*L. major*	1.66E-10	7.4	5.4	111
155	*L. major*	4.64E-07	4.2	6.56	69
212	*L. major*	5.51E-06	3.2	6.83	49
222	*L. major*	4.05E-12	2.2	5.9	46
172	*L. major*	4.58E-10	4.1	6.83	62
678	*L. tropica*	3.93E-06	2.5	6.13	113
533	*L. tropica*	0.012	1.8	5.93	114
52	*L. tropica*	0.003	2.1	5.91	102
719	*L. tropica*	2.90E-06	3.8	6.05	97
76	*L. tropica*	2.18E-04	5.6	4.24	95
701	*L. tropica*	2.92E-04	2.1	5.84	98
708	*L. tropica*	0.007	2	4.69	71
552	*L. tropica*	2.67E-07	4.7	5.53	98
453	*L. tropica*	6.08E-05	2	6.04	114
451	*L. tropica*	1.28E-04	2.6	5.96	114
130	*L. tropica*	6.12E-05	1.5	4.91	76
126	*L. tropica*	9.75E-06	2.1	4.35	77
546	*L. tropica*	0.005	2.5	5.59	91
508	*L. tropica*	1.55E-04	2.1	6.28	113
95	*L. tropica*	1.12E-06	9.1	4.39	88
741	*L. tropica*	1.16E-05	2.4	4.5	21

**ANOVA:** Analysis of variance test; **PI:** Isoelectric points; **MW:** Mulecular weight
